# Spin States of Trioxotriangulene Controlled by Si–O Bond Formation and Dissociation on AuSi_*x*_ Surfaces

**DOI:** 10.1021/acs.nanolett.5c03477

**Published:** 2025-08-13

**Authors:** Zhangyu Yuan, Toshikaze Kariyado, Tsuyoshi Murata, Kewei Sun, Donglin Li, Oscar Custance, Yasushi Morita, Shigeki Kawai

**Affiliations:** † Graduate School of Pure and Applied Sciences, University of Tsukuba, Tsukuba, Ibaraki 305-8571, Japan; ‡ Center for Basic Research on Materials, 52747 National Institute for Materials Science, Sengen 1-2-1, Tsukuba, Ibaraki 305-0047, Japan; § Research Center for Materials Nanoarchitectonics, National Institute for Materials Science, Namiki 1-1, Tsukuba, Ibaraki 305-0044, Japan; ∥ Department of Applied Chemistry, Faculty of Engineering, 12709 Aichi Institute of Technology, Yachigusa 1247, Yakusa, Toyota, Aichi 470-0392, Japan; ⊥ International Center for Young Scientists, National Institute for Materials Science, 1-2-1 Sengen, Tsukuba, Ibaraki 305-0047, Japan

**Keywords:** single molecule, neutral radical, spin, Si−O bond, scanning tunneling microscopy, tip-induced manipulation

## Abstract

The radical molecules have attracted significant attention from researchers because their electronic and spin properties can be controlled via the structure and heteroatoms. With the advancements in on-surface synthesis, it has become possible to conduct spin engineering at the single-molecule level. Here, we investigate the controllable polarized and electronic states of 4,8,12-trioxotriangulene adsorbed on AuSi_*x*_/Au­(111) surfaces with a combination of scanning tunneling microscopy (STM) operated at 4.3 K and density functional theory calculations. A rich variety of STM topographic contrasts of the molecule reveals various interactions between the molecule and the substrate. We found that the magnetic and electronic properties can be modulated through tip-induced formation and dissociation of a Si–O bond. This finding may pave the way for advancements in molecular spintronics.

## Graphical abstract

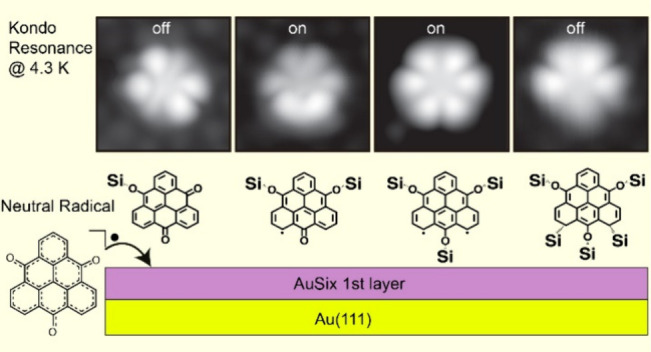

Triangulene has attracted tremendous attention from researchers since its first synthesis attempt in 1953.^1^ Unlike conventional polycyclic aromatic hydrocarbons, triangulene cannot be drawn with a Kekulé resonance structure. Such open-shell nanographene fragments not only have unique chemical and physical properties^2–8^ but also bear great potential for molecular spintronic devices.^9–11^ However, the triangulene synthesis in solution is in general challenging due to its high reactivity.^1^ Introducing sterically bulky substituents^12,13^ is one of the effective methods to obtain stable triangulene radicals in air. However, the bulky groups also reduce the intermolecular interaction, which may hinder the development of their application.^14^ By the introduction of three oxo groups, we synthesized and isolated highly air-stable 4,8,12-trioxotriangulene (TOT) without sterically bulky substituents.^15–17^ The 3-fold symmetric open-shell 25π-conjugated system is responsible for the high stability due to its delocalized electronic spin distribution with the largest spin density at the central carbon atom. Such properties are crucial for applications in energy conversion and storage,^18,19^ spin memories,^20,21^ near-infrared absorption,^22^ electrical conductors,^23–25^ and electrocatalyst for the oxygen reduction reaction.^26^ However, it is still unclear how the properties of TOT are modified by substrate adsorption and the chemical bonds at the single-molecule level.

The emergence of an on-surface synthesis strategy, which involves the bottom-up construction of molecular building blocks from small precursor molecules,^27^ has realized syntheses of unsubstituted,^28^ heterodoped,^29,30^ and π-extended^31–33^ triangulenes on various surfaces under ultrahigh-vacuum (UHV) conditions. The electronic and magnetic properties were also characterized in detail with scanning tunneling microscopy (STM) at low temperatures.^34–36^ Consequently, exotic spin-exchange coupling between the triangulene units has been investigated, in conjunction with successive tip-induced dehydration.^37^ Although the high reactivity may lead to further bond formation on the surface, modulating the electronic and magnetic properties of triangulene molecules by chemical bond formation is still scarce.

Here, we study the spin-polarized and electronic states of TOT molecules adsorbed on AuSi_*x*_/Au­(111) surfaces by low-temperature STM and scanning tunneling spectroscopy (STS). A rich variety of STM topographic contrast of TOT indicates the presence of various interactions between the molecule and the substrate. By applying pulse bias voltages and tip-induced manipulation, we could dissociate the Si–O bond and change the adsorption site in a controlled manner, resulting in modulations of the electronic and magnetic properties.

The TOT molecules (Figure 1a) were deposited on a AuSi_*x*_/Au­(111) surface (Figure S1), which was used as an electronic decoupling layer to study the electronic properties of graphene nanoribbons.^38,39^ Figure 1b shows the large-scale STM topography taken after the deposition of TOT on AuSi_*x*_/Au­(111) kept at 20 K. The substrate temperature was low enough so that individual molecules adsorbed homogeneously on both the first and second AuSi_*x*_ layers (Figure S2). We identified several types of TOT molecules, named types 1a, 1b, and 2–4, indicated by white, blue, yellow, red, and green arrows, respectively. Among these types, types 1–3 existed on both the first and second layers, whereas type 4 was only seen on the first layer (Figure 1c). To investigate the detailed structures of the TOT, close-up view STM topographies were recorded (Figure 1d–h). Notably, the appearance of type 1 on the first layer differs from that on the second one, which may relate to the different molecule–substrate interactions. Due to this difference in appearance, we further categorized type 1 on the first and second layers into type 1a (Figure 1d) and type 1b (Figure 1e), respectively. More structural contrast in the STM topography of type 1b results from the greater electronic decoupling property of the second layer than that of the first layer (see the arrows in Figure 1e). Types 1–3 (Figure 1d–g) had similar 3-fold symmetryies, while type 4 had a 2-fold symmetry (Figure 1h).

**Figure 1:**
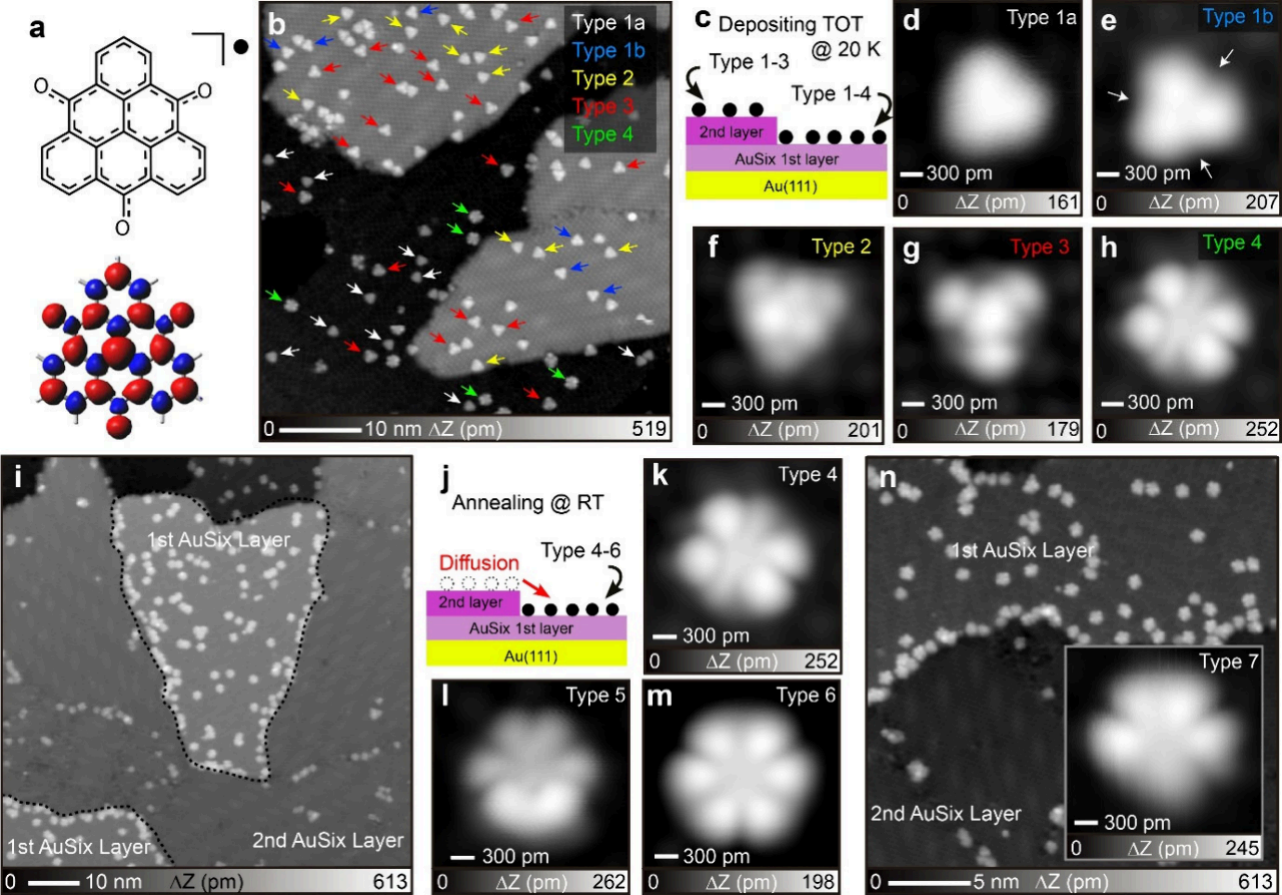
Formation of AuSi_*x*_ intercalation and deposition of TOT on AuSi_*x*_/Au­(111). (a) Chemical structure (upper panel) and electronic spin distribution (lower panel) of TOT. (b) Large-scale STM topography of the sample after deposition of TOT on AuSi_*x*_/Au­(111) kept at 20 K. Arrows indicate the four different types of TOT. (c) Schematic drawing of the TOT distribution on the first and second AuSi_*x*_ layers. Close-up views of type 1 on the first in panel d and second layers in panel e. (f–h) STM topographies of types 2–4 located on the first layer. (i) Large-scale STM topography after annealing the sample at RT for 15 min. The Moiré pattern is seen only on the second AuSi_*x*_ layer. (j) Schematic drawing of the sample after annealing at RT. (k–m) Close-view STM topographies of types 4–6 located on the first layer. (n) STM topography of the sample after further annealing at 338 K for 15 min. The inset shows a close-up view of type 7 located on the first layer. Measurement parameters: sample bias *V* = 200 mV and tunneling current *I* = 5 pA in panels b, h, k, and l, *V* = 200 mV and *I* = 10 pA in panels d, f, g, i, and n, *V* = 200 mV and *I* = 100 pA in panel e, and *V* = 100 mV and *I* = 50 pA in panel m.

After the sample was annealed at room temperature (RT) for 15 min, most of the molecules on the second layer moved to the first layer, on which the population of the molecule increased, as indicated by black lines (Figure 1i). The thermal diffusion of TOT to the first layer indicates that the molecule–substrate interaction on the second layer is smaller than that on the first one. Only a small amount of “bright dots” remained on the second layer, presumably related to molecules adsorbing at defect sites. Detailed STM images of TOT populations at the first and second layers are shown in Figure S3. Besides the diffusion of the molecule to the first layer (Figure 1j), we have found transformations of type 1–3 molecules to new forms, which are named as types 4–6. Figure 1k–m shows the corresponding close-up views of types 4–6, which no longer have a triangular shape. Figure 1n shows the large-scale STM topography taken after further annealing of the sample at 338 K for 15 min. Type 4–6 molecules eventually transformed into a new form, type 7, with a fish-like shape (inset of Figure 1n). An analysis of the apparent STM corrugations of each type can be found in Figure S4.

Next, STS measurements were conducted. We expected that all types of TOT adsorbed on the AuSi_*x*_ layer would have a *S* = 1/2 open-shell character, which should induce a Kondo resonance near the Fermi level. However, the short-range d*I*/d*V* curves measured above types 1–3 had no significant signal, related to the magnetic properties (Figure S5). In contrast, the long-range d*I*/d*V* curve of type 1a shows the highest occupied molecular orbital (HOMO) and lowest unoccupied molecular orbital (LUMO) states at −1.07 and 1.67 V, respectively (Figure 2a). Such clear molecular orbital states have been measured for pentacene adsorbed on a thin NaCl film formed on Cu(111),^40^ which is well-known as an electronic decoupling layer. A similar decoupling effect was measured for fullerene adsorbed on the AuSi_*x*_ first layer formed on Au(111),^41^ while the decoupling strength of the AuSi_*x*_ first layer was weaker than that of the ultrathin NaCl film. Thus, this spectrum indicates that type 1a was significantly decoupled from the gold substrate by AuSi_*x*_ intercalation, resulting in no significant interaction between the net spin of TOT and the conduction electrons of the substrate. We found that the adsorption sites of types 1b, 2, and 3 were frequently changed by applied voltages, which prevented stable long-range STS measurements. To investigate the structures of types 1–3, the tip apex was terminated by a CO molecule.^42^ The bond-resolved images show that the TOT molecules were intact, exhibiting the 3-fold symmetric structures and adsorbed flat on the surface (Figure 2b–e). Note that the asymmetric tip effect and tilt of the molecule can be seen in the bond-resolved images.

**Figure 2:**
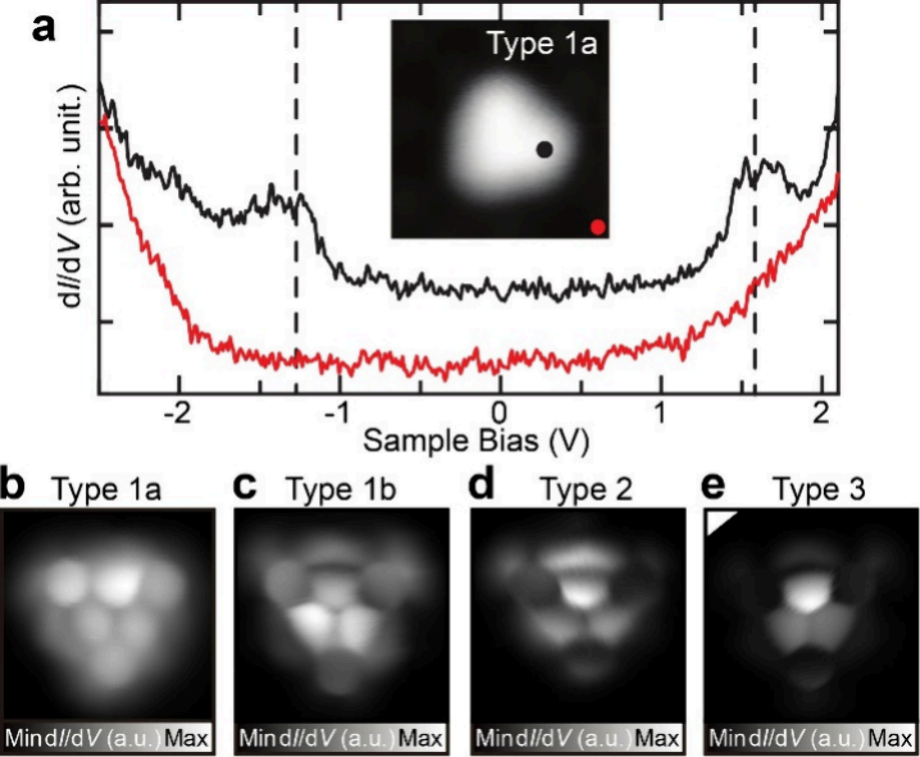
Electronic decoupling and bond-resolved imaging. (a) Long-range d*I*/d*V* curve of type 1a located on the first AuSi_*x*_ layer. The red curve was taken from the AuSi_*x*_ surface as a reference. The curves are shifted vertically for clarity. Set point of the gap: *V* = 200 mV and *I* = 100 pA. (b–e) Contant-height high-resolution d*I*/d*V* maps taken at *V* = 1 mV with *V*_ac_ = 10 mV. Type 1a was located on the first AuSi_*x*_ layer, while types 1b, 2, and 3 were located on the second layer.

In contrast to type 1a, distinct peaks appeared in the d*I*/d*V* curves taken above types 4–7, which were attributed to HOMO and LUMO as well as the singly occupied molecular orbital and singly unoccupied molecular orbital, as marked by dashed lines (Figure 3a–d). The absence of zero-bias peak indicates the closed-shell structures of types 4 and 7 (Figure 3a and d). In contrast, the d*I*/d*V* curves measured above types 5 and 6 have peaks around the Fermi level (Figure 3b and c and Figure S6). The sharp peak of type 5 indicates the existence of a localized unpaired electron spin, characterizing it as a *S* = 1/2 open-shell structure.^27,33,43–46^ In contrast, type 6 had a broadened asymmetric peak, which can be attributed to a *S* = 1 system.^27,45–49^ Two spins are ferromagnetically coupled.^50^ We also noticed a peak around 38 mV, indicated by the black dashed line in Figure S6b, which could be related to a molecular orbital. To investigate the presence of Kondo resonance, we conducted temperature-dependent measurements for both types 5 and 6 and found that both curves were broadened with an increasing temperature (Figure S7a and b). However, the temperature dependence of the peak broadening for type 6 was relatively weak. We attempted to fit the measured d*I*/d*V* curves with Frota (Kondo peak) and Gaussian (molecular orbital) functions as indicated by purple and blue curves in Figure S7b, respectively. By extracting the effective half width at half maximum (HWHM) values at each temperature (Figure S7c), we obtained a Kondo temperature of 171 K, which is somehow higher than those in previous studies with similar systems.^43–47^ We assume that the imperfect peak deconvolution is one of the reasons for such a high Kondo temperature. Nevertheless, types 5 and 6 possess *S* = 1/2 and 1 characters, respectively. The peaks of the d*I*/d*V* curves for both types 5 and 6 were significantly shifted from the Fermi level, and their magnitudes varied, depending on the measurement site on the molecule. We also attribute these shifts to the convolution of molecular orbital signals. The different spin states most likely arose from the bond formations between the oxygen atoms in the TOT molecule and the Si atoms of the substrate (Figure 3f and g). Since type 7 was obtained by higher temperature annealing, we deduced further bond formation between the unpaired electron sites and the Si atoms (Figure 3h). We tentatively assign a structure with one Si–O bond to type 4 (Figure 3e). The corresponding high-resolution images of types 4 and 7 are shown in Figure S8.

**Figure 3:**
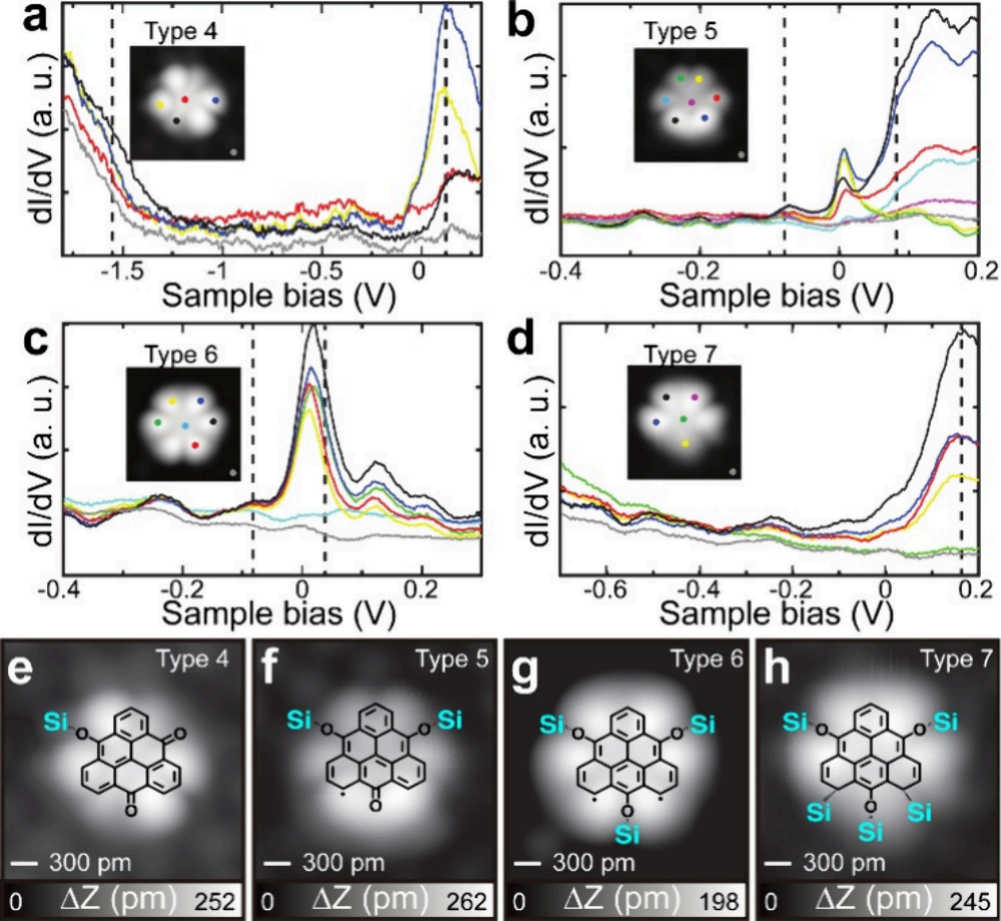
STS and magnetic fingerprints. (a–d) d*I*/d*V* curves measured above types 4–7 located on the first AuSi_*x*_ layer and (e–h) corresponding proposed chemical structures. Gray curves were taken on the AuSi_*x*_ surface as references. Measurement parameters: before each d*I*/d*V* curve measurement, the tip–sample gap was adjusted at *V* = −1.6 V, *I* = 100 pA, and *V*_ac_ = 10 mV in panel a, *V* = −400 mV, *I* = 100 pA, and *V*_ac_ = 10 mV in panels b and c, *V* = −600 mV, *I* = 100 pA, and *V*_ac_ = 10 mV in panel d, *V* = 200 mV and *I* = 5 pA in panels e and f, *V* = 100 mV and *I* = 50 pA in panel g, and *V* = 200 mV and *I* = 40 pA in panel h.

Tip-induced manipulation has been employed to reposition molecules^51^ and to induce single-molecule reactions,^52–59^ which can efficiently induce not only bond formation and dissociation^60^ but also configurational switching.^61^ We used this technique to prove our proposed chemical structures from an experimental perspective. We first positioned the tip above type 2 on the second AuSi_*x*_ layer and set the tip close to the molecule until the tunneling current increased to 6 nA, applying a bias voltage of 200 mV. Then, the tip was laterally moved along the dashed line (Figure 4a). After this manipulation, we found that type 2 was transformed to type 1b. Since the molecule on the first layer was manipulated with a smaller tunneling resistance compared to that on the second layer, we deduce that the interaction between the molecules and the substrate on the first layer is stronger than that on the second layer. Nevertheless, this lateral manipulation reveals that the various contrasts found in STM topographies (Figure 1) are related to different adsorption sites on the AuSi_*x*_ surface. We could not laterally manipulate types 4–7, which was most likely due to chemical bond formation between the oxo groups and the Si atoms at the surface. Thus, we next attempted to dissociate the bond between the molecule and the silicon atom by exciting the bond with a tunneling current at a given voltage. The tip was positioned above the molecule as indicated by a green dot in the inset of Figure 4b. The bias voltage was swept from 0 to −1.75 V while recording the tunneling current and then swept back to 0 V. After this process, type 7 was switched to type 6. On close inspection of the *I*–*V* curve, we found two events indicated by black arrows in Figure 4b, which are most likely related to the dissociation of two C–Si bonds. By repeating the voltage pulses, we were able to dissociate single Si–O bonds one by one and produce types 6, 5, and eventually 4. If the tip-induced manipulations were repeated, we finally obtained an intact TOT, that is, type 1 (Figure S9). This sequential dissociation strongly suggests that type 4 has one Si–O bond. We could also form one Si–O bond, synthesizing type 4 from type 1. However, multiple Si–O bond formation (types 5–7) was unsuccessful, which may be related to the adsorption geometry on the AuSi_*x*_ surface.

**Figure 4:**
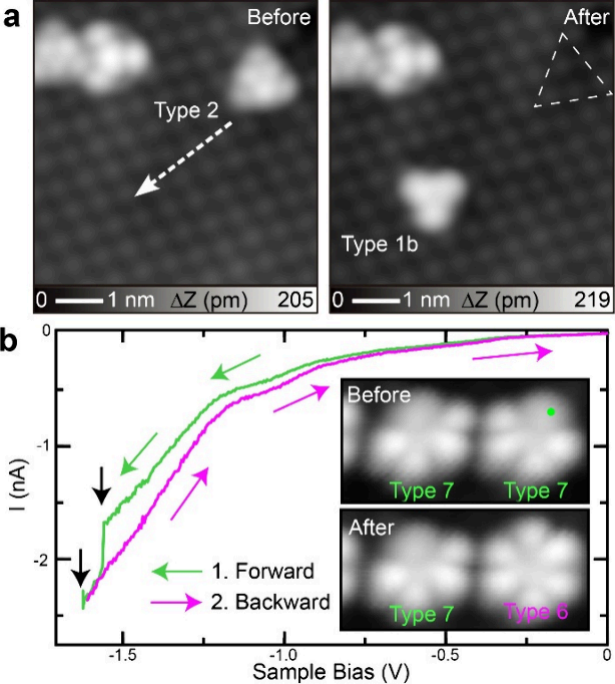
Tip-induced switching of different types of TOT. (a) Switching to type 1b from type 2 located on the second AuSi_*x*_ layer by a tip-induced lateral manipulation. (b) Tunneling curve versus sample bias (*I*–*V*) curve recorded during a switching event from type 7 to type 6 located on the first layer. Measurement parameters: *V* = 200 mV and *I* = 10 pA in panel a.

To understand the detected magnetic properties of TOT on the AuSi_*x*_ surface in detail, we conducted density functional theory (DFT) calculations for types 5 and 6 with the generalized gradient approximation (GGA) level using the OpenMX package.^62–64^ TOT was first adsorbed on the AuSi_*x*_ layer formed on Au(111). Based on the initial position of the molecule, various relaxed structures were obtained. Among them, we found configurations in which two and three oxygen atoms of TOT molecules were connected to Si atoms (Figure 5a and b), namely, types 5 and 6, respectively. As observed in the experiment, type 5 is significantly tilted as the height difference between the highest C atom and the lowest C atom is approximately 70 pm; in contrast, type 6 is almost planar. In both cases, the Si–O bonds induce local deformations in the molecule. Using these relaxed structures, the partial density of states (pDOS) accumulated within the range between the Fermi energy and the bias potential were calculated. The STM topographies of types 5 and 6 were also simulated by inspecting the shape of the isosurfaces of pDOS, using the Critic2 package (Figure 5c and d).^65,66^ We found that the simulated results are in agreement with the experiments. To obtain the spin density distributions, spin GGA calculations were conducted (Figure 5e and f). Clearly, the spin densities resembled those of the isolated molecules corresponding to types 5 and 6. We also calculated the spin density of types 5 and 6 in vacuum at different levels (Figures S10–S13). To represent the Si–O bond, −SiH_3_ groups were connected to the oxygen atoms of the molecule. We found that the distribution of spin densities for the molecules on the AuSi_*x*_ surface and in a vacuum was almost the same, indicating that the AuSi_*x*_ substrate has only a marginal effect on the local spin distribution. The calculated spin magnetic moments are ∼0.66 μ_B_ and ∼1.49 μ_B_ for types 5 and 6, respectively. The deviation from the values of the isolated molecules (1 and 2 μ_B_) most likely results from the weak coupling between the molecular orbitals and the metallic states of the Au slab. Due to the coupling, molecular orbital energies are effectively smeared and partial filling of majority/minority spin states becomes possible. Our Mulliken population analyses indicate that the charge transfers to types 5 and 6 from the surface are 0.38 and 0.50 e, respectively. These excess charges fill the minority spin state, leading to partial cancellation of the spin magnetic moment. We understood that optimizing the Si density in the AuSi_*x*_ layer would increase the consistency of the spin magnetic moments; however, due to the high computational cost, a thorough investigation of the silicon density is beyond our focus. Nevertheless, our DFT calculations indicate that the formation of the Si–O bond plays a decisive role in the magnetic properties of TOT.

**Figure 5:**
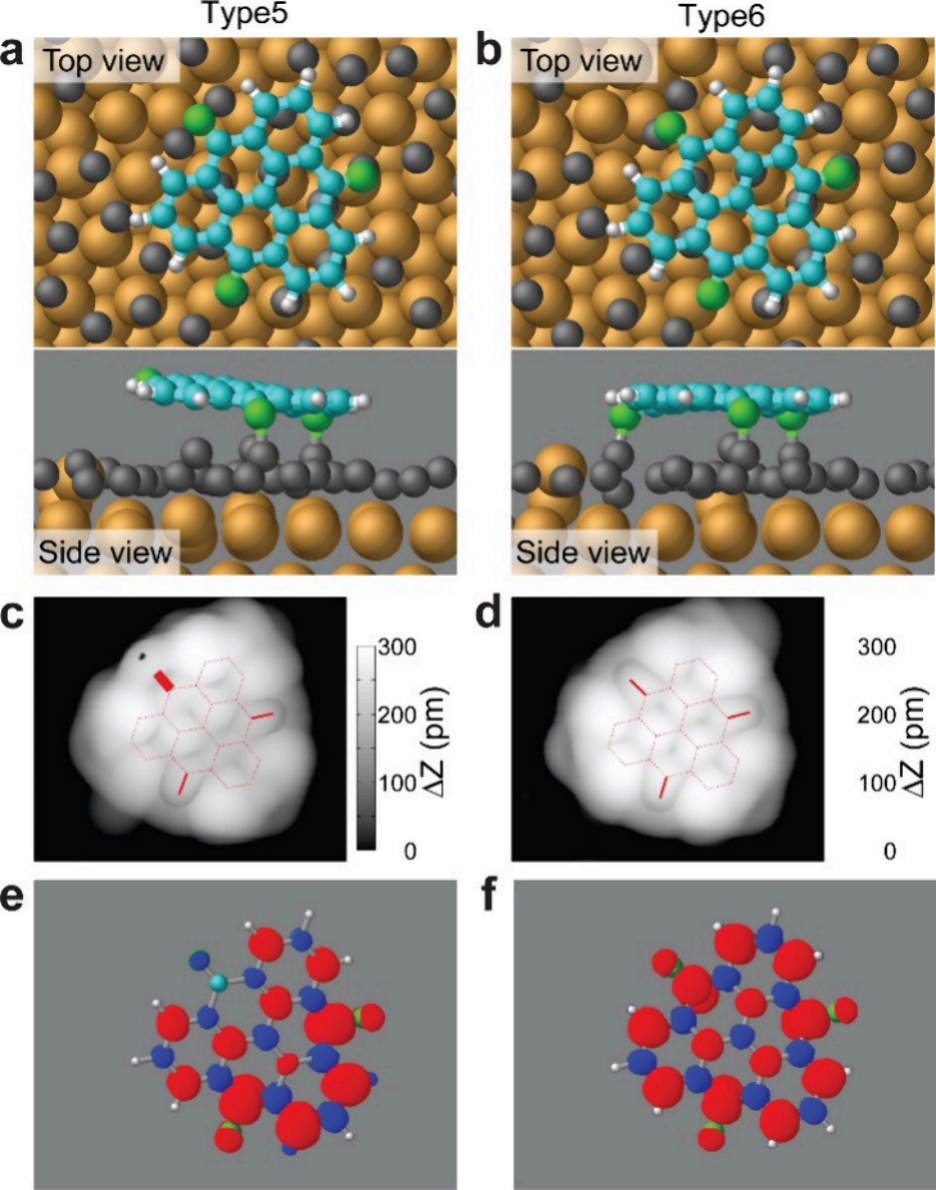
STM contrast and spin density obtained from first-principles calculations. (a and b) Relaxed structures of types 5 and 6 adsorbed on the first AuSi_*x*_ layer on Au(111), calculated using DFT with the GGA level: (upper panel) top view and (lower panel) side view. White, cyan, green, gray, and yellow balls represent hydrogen, carbon, oxygen, silicon, and gold atoms, respectively. (c and d) Corresponding simulated STM topographies and (e and f) spin density maps of types 5 and 6. The arrows indicate that the Si atoms are connected to the O atom.

In conclusion, we investigated the spin polarization of TOT on the AuSi_*x*_/Au­(111) surface and demonstrated that the magnetic and electronic properties can be controlled by the surface and chemical bonding. The rich variety of STM topography contrasts was observed by annealing the sample at different temperatures. We found that the magnetic and electronic properties of the molecule are significantly affected by molecular adsorption sites and the formation of Si–O bonds between the oxo group and the surface silicon atom. We expect that the Si–O bond formation can be achieved using TOT derivatives.^67^ Furthermore, these properties can be switched by tip-induced formation and dissociation of the Si–O bonds as well as lateral manipulation of the molecule in a controlled manner. We believe that the ability to control the chemical bonding of radical molecules by the tip may pave the way for advancements in molecular spintronics.

## Supplementary Material



## Notes

Detailed experimental sections, detailed theoretical calculation sections, formation of AuSi_*x*_/Au­(111), as-deposited molecules at 20 K, molecules after annealing at room temperature, line profiles of types 1–7, short-range d*I*/d*V* curves of types 1a, 1b, 2, and 3, short-range d*I*/d*V* curves of types 4 and 5, temperature dependence of the Kondo peak, high-resolution STM topographies and d*I*/d*V* maps of types 4–7, tip-induced switching of different types of TOT, spin density distribution maps calculated in the gas phase, calculation of the radical dianion and diradical trianion, calculation of the triangulene diradical, and line profiles of type 6 (PDF)
