# Supplementary material for: Spin States of Trioxotriangulene Controlled by Si–O Bond Formation and Dissociation on AuSi x Surfaces
Source: Nano Lett. 2025 Aug 13;25(34):13040–6. doi: 10.1021/acs.nanolett.5c03477 (PMC12395471; doi:10.1021/acs.nanolett.5c03477)
Supplement: Supplementary file 1 [file nl5c03477_si_001.pdf]

Supporting Information for

Spin States of Trioxotriangulene Controlled by Si-O Bond Formation and Dissociation on $AuSi_x$ Surfaces

Zhangyu Yuan[1,2], Toshikaze Kariyado[3], Tsuyoshi Murata[4], Kewei Sun[2,5], Donglin Li[2], Oscar Custance[2], Yasushi Morita[4]* and Shigeki Kawai[1,2]*

[1] *Graduate School of Pure and Applied Sciences, University of Tsukuba, Tsukuba 305-8571, Japan.*

[2] *Center for Basic Research on Materials, National Institute for Materials Science, Sengen 1-2-1, Tsukuba, Ibaraki 305-0047, Japan.*

[3] *Research Center for Materials Nanoarchitectonics, National Institute for Materials Science, Namiki 1-1, Tsukuba 305-0044, Japan.*

[4] *Department of Applied Chemistry, Faculty of Engineering, Aichi Institute of Technology, Yachigusa 1247, Yakusa, Toyota, Aichi, 470-0392, Japan.*

[5] *International Center for Young Scientists, National Institute for Materials Science, 1-2-1 Sengen, Tsukuba, Ibaraki 305-0047, Japan.*

STM experiment:

The experiments were conducted by a homemade low temperature STM system operating at 4.3 K under ultrahigh vacuum condition (< 1×10^{-10} mbar). A clean Au(111) surface was prepared by several cycles of Ar^+ sputtering for 10 min and annealing at 703 K for 15 min. Si atoms were evaporated by a one-pocket electron beam evaporator (SPECS GmbH) and deposited on a clean Au(111) surface at 423 K to form the $AuSi_x$/Au(111) surface. 4,8,12-trioxotriangulene (TOT) molecules were deposited on the $AuSi_x$/Au(111) substrate kept at 20 K. A tungsten tip made by chemical etching was used for STM and STS measurements. The STS measurements were conducted at constant tip height using lock-in techniques. The modulation zero-to-peak amplitude of the bias voltage was 10 mV with a frequency of 510 Hz.

Theoretical calculations

The calculation follows the next scheme: (i) structural relaxation of a finite thickness Au slab with (111) surface, (ii) structural relaxation with randomly distributed Si atoms on top of the Au slab, and (iii) structural relaxation and electronic/magnetic structure analysis with TOT molecule. In our setup, the Au slab is five-atom thick perpendicular to the (111) surface and has 6×6 hexagonal supercell parallel to the (111) surface. The slab is placed in a hexagonal unit cell with z-axis length being 3.6 nm to have a sufficient vacuum layer above the slab. The in-plane lattice constant for this $6 \times 6 \times 5$ layer is set to 1.75 nm. We included 36 Si atoms in this unit cell, in line with the conditions used in a previous study.[39] In order to handle a large unit cell, we use OpenMX package,[61–63] in which a localized basis method is implemented. For H, C, O, Si, and Au atoms, the basis orbitals are chosen to be of types s2p2d1, s3p2d2, s3p2d2, s3p3d2, and s3p3d2f1, respectively. To account for the exchange-correlation functional, we have employed PBE-GGA.Van der Waals interaction is included through the DFT-D3 method. The spin-orbit coupling has been neglected for simplicity. The reciprocal space integrations were done on 2×2×1 k-point meshes. In each self-consistent step, the occupation of Kohn-Sham orbitals are controlled by the Fermi distribution function with a temperature of 600 K for structural relaxations and 300 K for electronic and magnetic structure analysis. The convergence thresholds for the self-consistent-field calculations and the structural relaxation are set to 1×10^{-7}

Hartree for the eigenenergy deviation, and 2×10^{-4} Hartree/Bohr for the maximum remaining force, respectively.

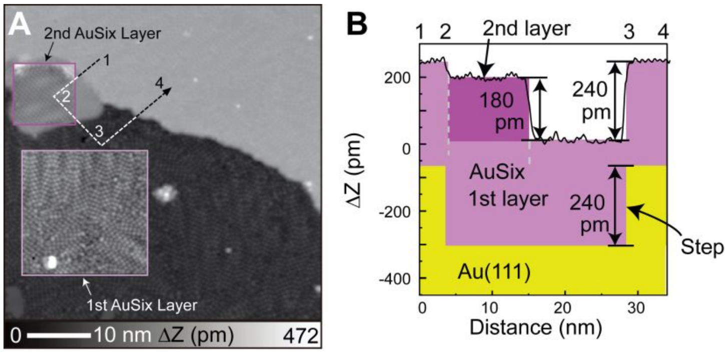

Figure S1. Formation of $AuSi_x$/Au(111). (A) Scanning tunneling microscopy (STM) topography of the $AuSi_x$/Au(111) surface. The inset shows an atomic resolution image of the second $AuSi_x$ layer over the area indicated by a pink square. A close-up view of the first $AuSi_x$ layer shows that the surface structure is almost identical to that of the $Au_{2.43}Si$ surface.[S1] We also found the formation of a Moiré pattern only on the second layer.[S21] (B) Line profile taken along the dashed lines indicated in (A). The height difference between the first and second layers, which were formed on the same Au(111) terrace, was approximately 180 pm. The first $AuSi_x$ layer marked in bright pink was higher than the second one marked in dark pink as shown in the line profile (inset of Figure 1A), which is related to the monoatomic step of the underlying Au(111) surface marked by yellow. Measurement parameters: V = 200 mV and I = 20 pA in (A).

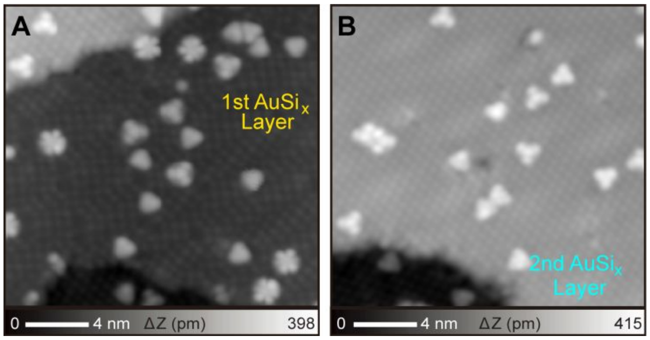

Figure S2. As-deposited molecules at 20 K. (A) Enlarged STM topographies measured on the first and (B) second $AuSi_x$ layers. Measurement parameters: V = 200 mV and I = 10 pA in (A) and (B).

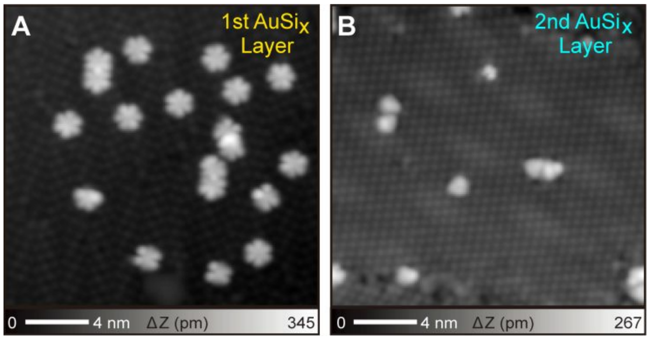

Figure S3. Molecules after annealing at room temperature. (A) STM topographies measured on the first and (B) second $AuSi_x$ layers after annealing the sample at room temperature for 15 min. Measurement parameters: $V = 200$ mV and $I = 10$ pA in (A) and (B).

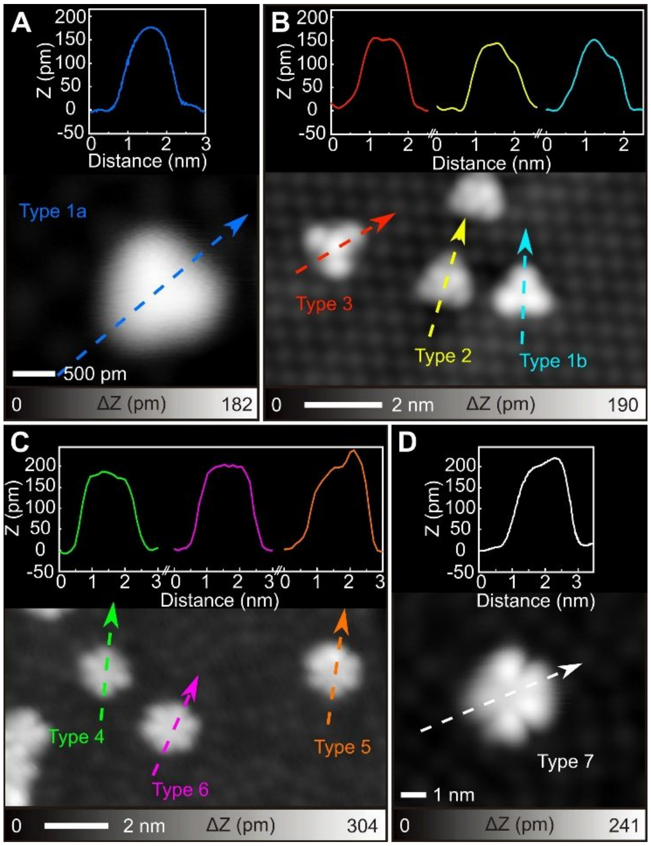

Figure S4. Line profiles of Type 1-7. Measurement parameters: $V = 200$ mV and $I = 100$ pA in (A), $V = 200$ mV and $I = 10$ pA in (B), (C) and (D).

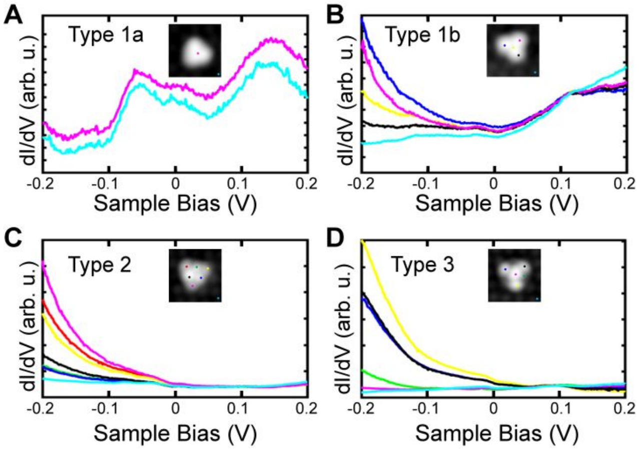

Figure S5. Short-range dI/dV curves of Type 1a, 1b, 2 and 3. Measurement parameters: V = 200 mV and I = 100 pA, V_{ac} = 10 mV in (A), V = 200 mV and I = 60 pA, V_{ac} = 1 mV in (B) and (C), V = 200 mV and I = 90 pA, V_{ac} = 10 mV in (D).

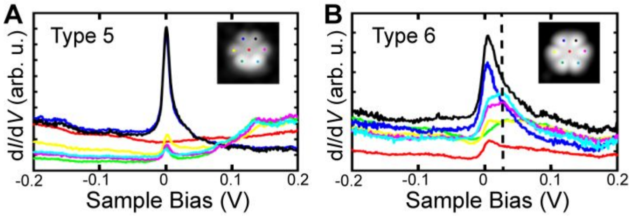

Figure S6. Short-range dI/dV curves of Type 4 and 5. (A) Short-range dI/dV curves of Type 5 and (B) Type 6. Measurement parameters: V = 200 mV and I = 70 pA, V_{ac} = 5 mV in (A), V = 200 mV and I = 160 pA, V_{ac} = 5 mV in (B).

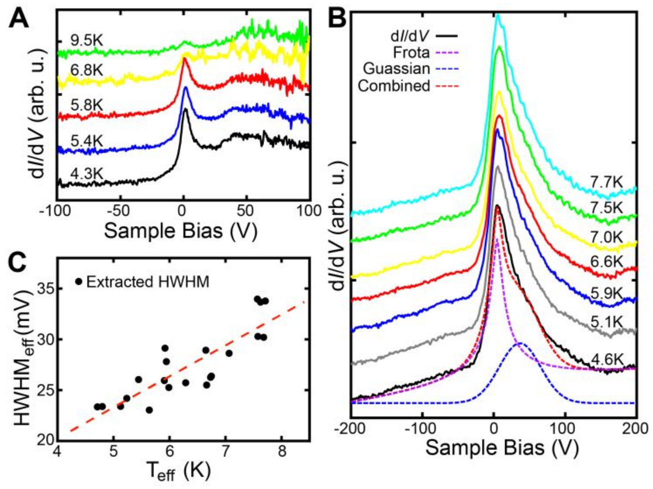

Figure S7. Temperature dependence of the Kondo peak. (A) d*I*/d*V* curves of Type 5 recorded at different temperatures. (B) d*I*/d*V* curves of Type 6 recorded at different temperatures. A Frota fit, a Gaussian fit, and combination of Frota and Gaussian fits for the black d*I*/d*V* curve are shown as purple, blue and red dashed lines, respectively. (C) Effective half-width at half-maximum (HWHM) of the Kondo peaks for Type 6 extracted from the Frota fits as a function of the temperature, together with the fitting curve in red. Measurement parameters: $V = 200$ mV and $I = 300$ pA, $V_{ac} = 3$ mV in (A) and (B).

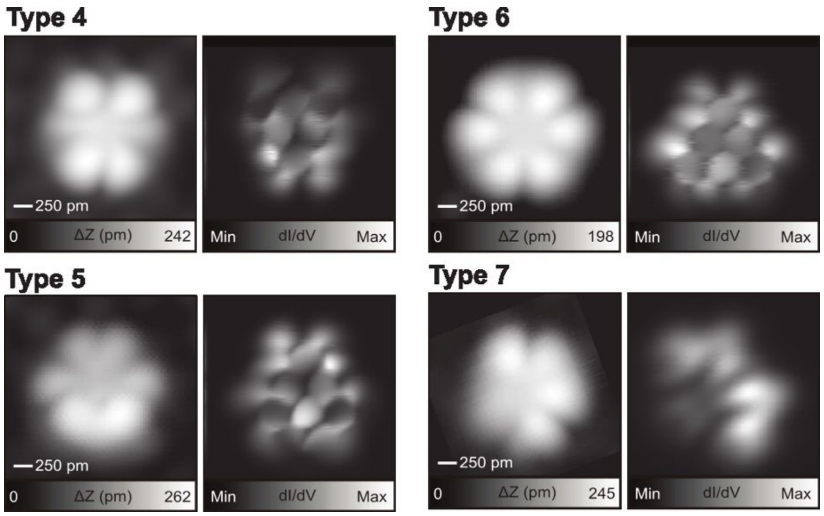

Figure S8. High-resolution STM topographies (left) and d*I*/d*V* maps (right) of Type 4–7. Measurement parameters: $V = 200$ mV and I = 5 pA in left STM image of Type 4 and 5, $V = 100$ mV and $I = 50$ pA in left STM image of Type 6, $V = 200$ mV and $I = 10$ pA in left STM image of Type 7, $V = 0.1$mV and $V_{ac} =$ 1 mV in right STM image of Type 4–7.

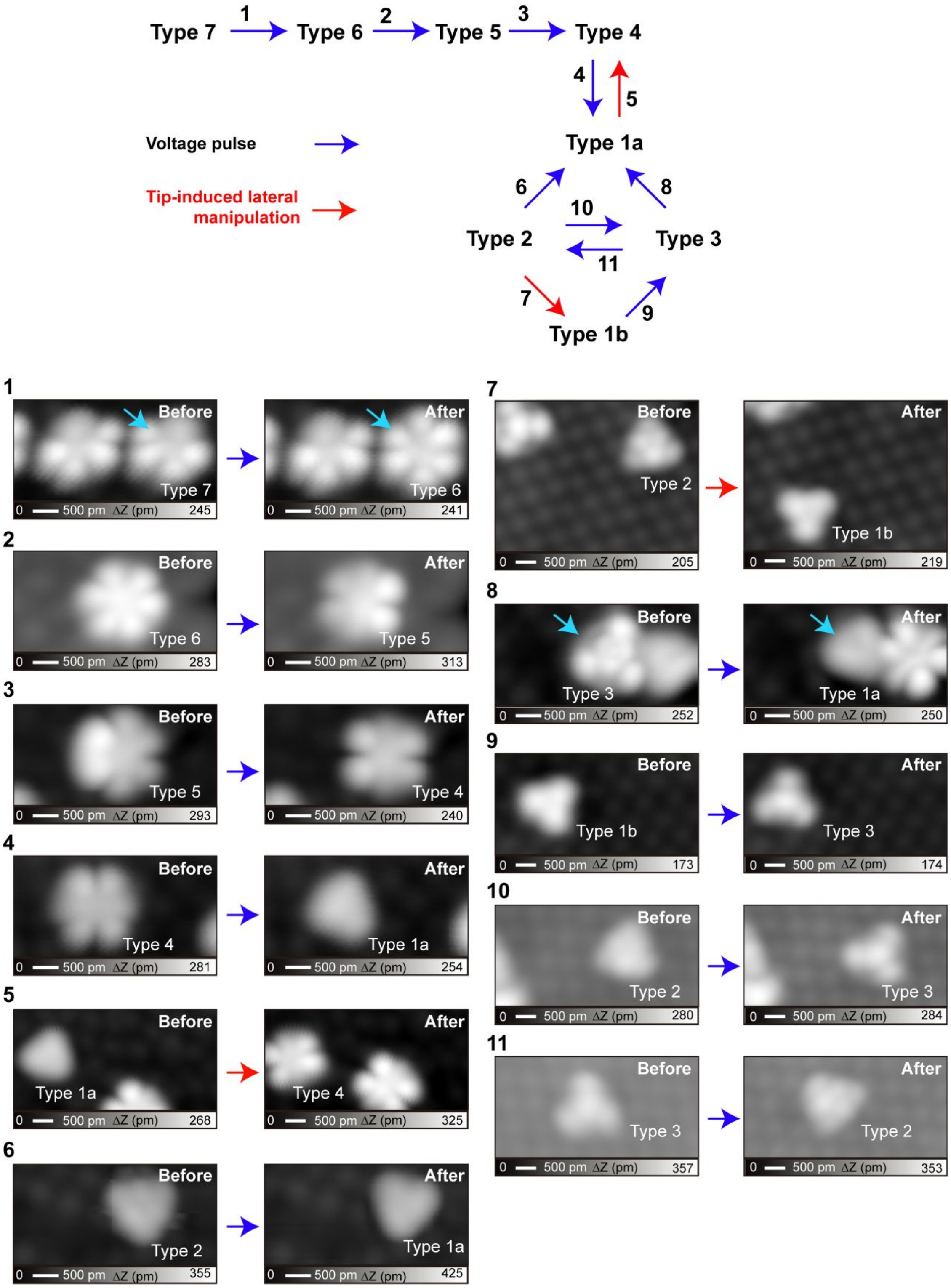

Figure S9. Tip-induced switching of different types of TOT by applying bias voltage pulses (blue arrows) and tip-induced lateral manipulations (red arrows). Measurement parameters: $V = 200$mV and $I = 10$pA.

We conducted density functional theory calculations for Type 5 and 6 with UB3LYP/6-311G(d,p) level. The SiH_3 groups were added to the oxygen sites to virtually represent TOT species in the experiment. Figures S10A and B show the spin density distribution of Type 5 and 6 molecules, respectively. We found that the Type 5 molecule has an $S = 1/2$ spin with a two-fold symmetry spin density distribution. On the other hand, the spin quantum number of Type 6 becomes an $S = 1$, and its spin density distribution has a three-fold symmetry; which are in agreement with the on-surface calculation (Figures 5E,F) The spin density distribution of Type 5 and 6 are also similar to those of the free TOT radical dianion and diradical trianion species (Figure S11), which relates to the doubly degenerate LUMOs of TOT.[S3,S4] In addition, the spin density distribution of Type 6 resembles that of the triangulene diradical (Figure S12). However, we found that the experimental constant-height d*I*/d*V* maps of Type 5 and 6 measured at singly occupied molecular orbital (SOMO) and singly unoccupied molecular orbital (SUMO) levels (Figures S10B,E) did not match the corresponding DFT-calculated ones (Figures S10C,F). Since two sets of Si-O bonds significantly tilted Type 5 on the $AuSi_x$ surface (Figures 5A,B), the topographic effect was dominant in the constant-height d*I*/d*V* map. Type 6 had three sets of Si-O bonds, which do not tilt the molecule when three Si sites are commensurate with the oxygen sites in the molecule (Figure 5B). However, the line profiles of Type 6 show a nonplanar molecular configuration (Figure S13), which is most probably due to the local deformation by the Si-O bonds. Thus, the agreement between the experimental constant-height d*I*/d*V* map and the corresponding calculations was rather limited due to the absence of the surface.

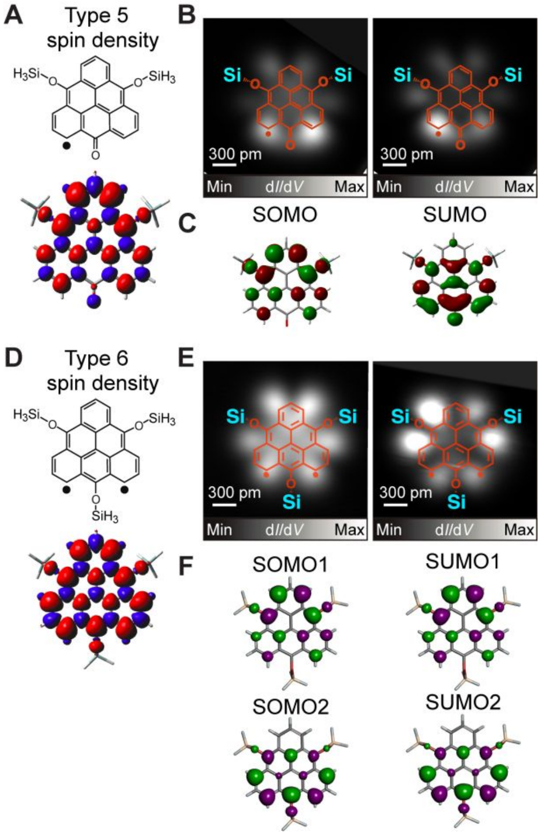

Figure S10. Spin density distribution maps calculated in gas phase. (A) Canonical chemical structures and spin density distribution maps calculated with UB3LYP/6-311G(d,p) level, of Type 5 and (B) Type 6. (C) Experimental constant-height d*I*/d*V* maps of Type 5 at SOMO/SUMO energies (set point at the gap: V = –95 mV and V_{ac} = 10 mV for the SOMO, V = 95 mV and V_{ac} = 10 mV for the SUMO). (D) DFT calculated local density of states SOMO/SUMO for Type 5. (E) Experimental constant-current d*I*/d*V* maps for Type 6 at SOMO/SUMO energies (set point at the gap: V = –90 mV and V_{ac} = 10 mV for the SOMO, V = 20 mV and V_{ac} = 10 mV for the SUMO). (F) DFT calculated local density of states SOMO/SUMO for Type 6.

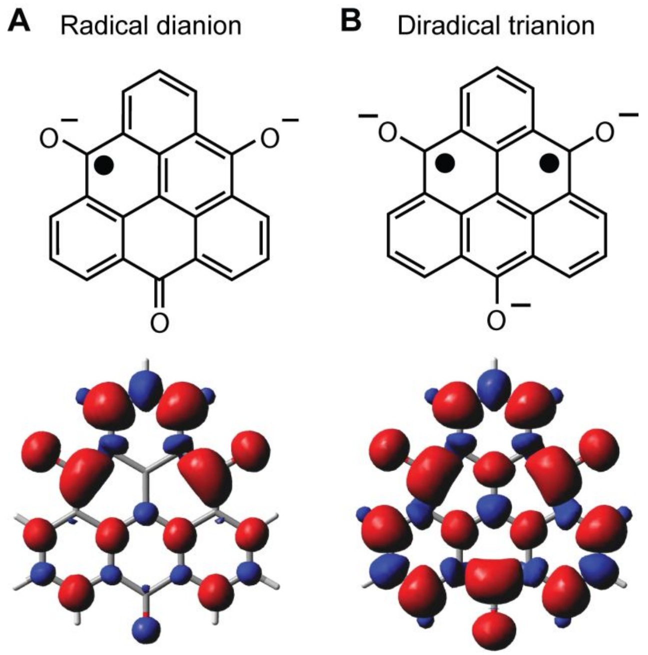

Figure S11. (A) Canonical Chemical Structures and spin density distribution maps of the radical dianion and (B) the diradical trianion.

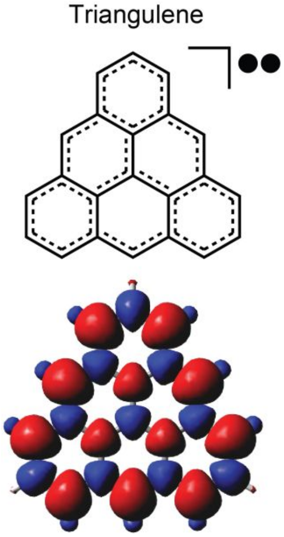

Figure S12. Canonical Chemical Structure and spin density distribution map of the triangulene diradical.

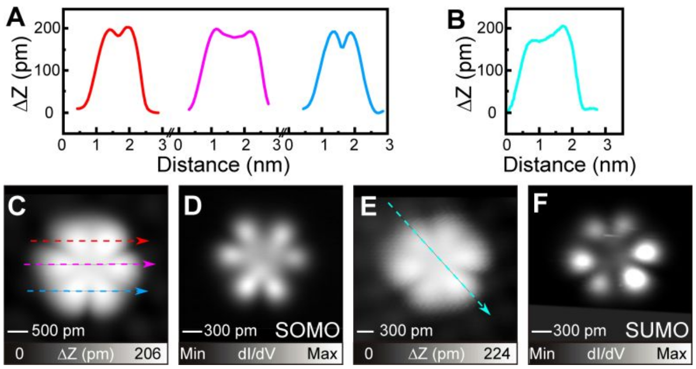

Figure S13. Line profiles of Type 6. (A) Line profiles taken along red, pink and blue dashed line, indicted in C. (B) Line profile taken along bright blue line indicated in E. (C) STM topography, (D) dI/dV map taken at the SOMO energy, (E) STM topography, and (F) dI/dV map taken at the SUMO energy. Set point of the gap: V = 200 mV and I = 10 pA in (C) and (E), V = –90 mV and V_{ac} = 10 mV in (D), V = 20 mV and V_{ac} = 10 mV in (F).

References

S1. Green, A. K.; Bauer, E. Silicide Surface Phases on Gold. *J. Appl. Phys.* **1981**, *52*, 5098−5106.

S2. Sadeddine, S.; Enriquez, H.; Bendounan, A.; Das, P. K.; Vobornik, I.; Kara, A.; Mayne,A. J.; Sirotti, F.; Dujardin, G.; Oughaddou, H. Compelling Experimental Evidence of a Dirac Cone in the Electronic Structure of a 2D Silicon layer. *Sci. Rep.* **2017**, *7*, 44400.

S3. Allinson, G.; Bushby, R. J.; Paillaud, J.-L.; Oduwole, D.; Sales, K. ESR Spectrum of a Stable Triplet π Biradical: Trioxytriangulene, *J. Am. Chem. Soc.* **1993**, *115*, 2062−2064.

S4. Allinson, G.; Bushby, R. J.; Paillaud, J.-L.; Thornton-Pett, M. Synthesis of a Derivative of Triangulene; the First Non-Kekulé Polynuclear Aromatic, *J. Chem. Soc.; Perkin Trans. 1* **1995**, *4*, 385−390.
